# Chinese Expert Consensus on Integrated Chinese and Western Medicine for the Treatment of Tic Disorders

**DOI:** 10.1111/jebm.70012

**Published:** 2025-04-16

**Authors:** Rong Ma, Sumei Wang, Yi Zheng, Jian Chen, Wenxiong Chen, Xia Cui, Yonghua Cui, Yanzhao Guo, Fei Han, Xinmin Han, Daohan Wang, Fan He, Ping Rong, Yan Hu, Yi Huang, Xiaoyan Ke, Bo Li, Min Li, Ruiben Li, Huanzhong Liu, Jing Liu, Zhisheng Liu, Xuerong Luo, Bingxiang Ma, Ping Shi, Jiaxin Sun, Hua Wang, Liping Wu, Min Wu, Haihong Yan, Baoqing Zhang, Jinsong Zhang, Junhua Zhang, Xilian Zhang, Xin Zhang, the Standard Research Project Group on Prevention and Treatment Strategies of Integrated Chinese and Western Medicine

**Affiliations:** ^1^ Department of Pediatrics First Teaching Hospital of Tianjin University of Traditional Chinese Medicine Tianjin China; ^2^ Department of Pediatrics, Dongfang Hospital Beijing University of Chinese Medicine Beijing China; ^3^ Department of Pediatric Psychiatry, Beijing Anding Hospital Capital Medical University Beijing China; ^4^ Hospital Management Office Zhejiang University of Chinese Medical Zhejiang China; ^5^ Department of Behavioral Development Guangzhou Women and Children's Medical Center Guangzhou China; ^6^ Department of Pediatrics Third Affiliated Hospital of Beijing University of Chinese Medicine Beijing China; ^7^ Department of Psychiatry, Beijing Children's Hospital Capital Medical University Beijing China; ^8^ Ten Departments of Encephalopathy Xi 'an Encephalopathy Hospital of Traditional Chinese Medicine Xi'an China; ^9^ Department of Pediatrics, Guang'anmen Hospital China Academy of Chinese Medical Sciences Beijing China; ^10^ Department of Pediatrics Jiangsu Provincial Hospital of Traditional Chinese Medicine Nanjing China; ^11^ Department of Psychiatry West China Hospital of Sichuan University Chengdu China; ^12^ Children's Psychohygiene Research Center The Affiliated Brain Hospital of Nanjing Medical University Nanjing China; ^13^ Department of Gastroenterology, Beijing Hospital of Traditional Chinese Medicine Capital Medical University Beijing China; ^14^ Department of Traditional Chinese Medicine Children's Hospital of Capital Institute of Pediatrics Beijing China; ^15^ Department of Psychiatry Chaohu Hospital of Anhui Medical University Chaohu China; ^16^ Department of Pediatrics Peking University Sixth Hospital Beijing China; ^17^ Department of Pediatric Neurology Wuhan Maternal and Child Health Hospital Wuhan China; ^18^ Department of Psychiatry The Second Xiangya Hospital of Central South University Changsha China; ^19^ Children's Brain Disease Diagnosis and Rehabilitation Center The First Affiliated Hospital of Henan University of TCM Zhengzhou China; ^20^ Department of Psychology Tianjin Children's Hospital Tianjin China; ^21^ Department of Pediatric Neurology Shengjing Hospital of China Medical University Shenyang China; ^22^ Department of Pediatrics Affiliated Hospital of Gansu University of Chinese Medicine Lanzhou China; ^23^ Department of Psychology Xinhua Hospital Affiliated to Shanghai Jiao Tong University of Medicine Shanghai China; ^24^ Department of Traditional Chinese Medicine Children's Hospital of Shanxi Province Taiyuan China; ^25^ Department of Pediatrics Affiliated Hospital of Shangdong University of Chinese Medicine Jinan China; ^26^ Evidence‐based Medicine Center Tianjin University of Traditional Chinese Medicine Tianjin China

**Keywords:** expert consensus, integrated Chinese and Western medicine, tic disorders, traditional Chinese medicine

## Abstract

**Objective:**

Tic disorders are neurodevelopmental conditions that manifest in childhood or adolescence and can significantly impact the quality of life of affected children and their families to varying degrees. Integrated traditional Chinese and Western medicine treatment strategies have demonstrated more pronounced efficacy and better safety profiles. However, there is currently no standardized clinical expert consensus on this approach. To address this, the National Administration of Traditional Chinese Medicine initiated a project, and the China Association of Chinese Medicine assembled a team of authoritative domestic experts to develop this expert consensus, aiming to provide practical and feasible integrated treatment strategies for clinical practice.

**Methods:**

This consensus identified clinical issues through research, conducted literature reviews, and established evidence based on systematic evaluations. Expert surveys, two rounds of Delphi questionnaires, and expert consensus meetings were conducted to formulate a series of recommendations.

**Results:**

We established a multidisciplinary consensus development panel. Based on systematic literature reviews, Delphi questionnaires, and consensus meetings, ten clinical issues were identified. Ultimately, a series of recommendations were developed, considering the balance of benefits and risks, the certainty of evidence, clinical feasibility, accessibility, and clinical acceptability.

**Conclusions:**

These recommendations comprehensively address key issues in the field of integrated traditional Chinese and Western medicine treatment, including indications for the use of Chinese or Western medicine alone or in combination, specific treatment protocols, methods for dose reduction and discontinuation, evaluation intervals, and the management of adverse reactions.

## 1 Introduction

Tic disorders (TD) are neurodevelopmental disorders that arise during childhood or adolescence, and they are characterized by involuntary, purposeless, rapid, repetitive, and stereotyped muscle movements in one or multiple parts of the body and/or vocal tics [1, 2, 3]. Epidemiological studies on mental disorders among children and adolescents in China revealed that the prevalence of TD among students aged 6–16 years was 2.5%, with transient TD, chronic TD, and Tourette syndrome (TS) accounting for 1.2%, 0.9%, and 0.4%, respectively [4, 5]. Because of limited awareness of TD symptoms among parents, some children may remain undiagnosed, thus the actual prevalence of TD may be higher than reported. Approximately 50% of children with TD suffer from one or more comorbid mental disorders, adding to the complexity and severity of the condition [6, 7].

The objectives of traditional Chinese medicine (TCM) and Western medicine in treating TD are similar in that both aim to alleviate tic symptoms and comorbid conditions, thereby improving the quality of life [8]. In China, the clinical application of integrated Chinese and Western medicine for managing TD is quite prevalent, with an increasing research focus. Studies have shown that the clinical efficacy of integrated Chinese and Western medicine treatment is superior to single treatment regimens, with fewer adverse reactions [9].

Nevertheless, no clinical consensus or guidelines have been available for preventing and treating TD using approaches integrating Chinese and Western medicine [10].

## 2 Methods

### 2.1 Scope and Target Population of the Consensus

This consensus includes guidelines for disease diagnosis, treatment (Western medicine, TCM, and integrated Chinese and Western medicine), and prevention and care. Current guidelines or consensus statements cover the disease diagnosis and treatments using Western and Chinese medicine; therefore, this document focuses on clinical issues and recommendations for treatment using integrated Chinese and Western medicine.

The consensus is intended for TCM practitioners, practitioners of integrated Chinese and Western medicine, and Western medicine practitioners willing to prescribe TCM in the departments of pediatrics and psychiatry (psychology). The target population is patients under 18 years old diagnosed with TD.

### 2.2 Formation of the Consensus Expert Group

The expert team consisted of three project leaders (two TCM leaders and one Western medicine leader), a consensus expert group (15 TCM experts, 10 Western medicine experts, and 2 methodology experts), and a secretariat (two TCM members and one Western medicine member). The consensus expert group was drawn from 15 provinces and cities across China.

### 2.3 Declaration and Management of Conflicts of Interest

The development of the consensus, funded by the China Association of Chinese Medicine, was not associated with potential conflicts of interest. To prevent conflicts of interest during the development, all members involved in drafting the consensus signed a conflict‐of‐interest statement before official participation, acknowledging the absence of any commercial, professional, or other interests associated with the subjects covered by this consensus as well as any interests that were possibly affected by the outcomes of the consensus.

### 2.4 Consensus Registration

The consensus was registered on the International Practice Guideline Registry Platform (http://guidelinesregistry.cn/; registration no.: PREPARE‐2023CN100).

### 2.5 Formulation of Clinical Questions

Based on existing domestic and international guidelines and consensus, clinical research, systematic reviews, and meta‐analyses on TCM, Chinese herbal medicine, Western medicine, and pharmacotherapy for TD, a compilation of clinical questions was drafted, and a survey questionnaire was developed. Subsequently, through one round of clinical survey and two rounds of expert discussions, the collection, screening, and supplementation of clinical questions were completed, resulting in an initial set of 51 clinical questions. After two rounds of Delphi questionnaires and one expert consensus meeting, ten clinical questions related to integrated Chinese and Western medicine were finalized.

### 2.6 Evidence Retrieval, Evaluation, and Comprehensive Analysis

#### 2.6.1 Literature Search

A detailed search strategy and inclusion/exclusion criteria were devised based on the proposed clinical questions. Chinese literature was searched in the China National Knowledge Infrastructure (CNKI), Chinese Science and Technology Journal Database (VIP), Wanfang Database (WANFANG), and Chinese Biomedical Literature Database (CBM). English literature was searched using PubMed, Embase, Web of Science, and Cochrane Library. In addition, manual searches were conducted, and references from relevant literature were supplemented. The search period encompassed the establishment of the databases up to September 2023.

#### 2.6.2 Literature Screening and Data Extraction

NoteExpress literature management software was used to manage and initially screen the literature, focusing on guidelines and consensus, systematic reviews, and original studies that met the inclusion and exclusion criteria. Full‐text articles were reviewed to extract relevant data, which mainly included authors, publication date, study design, study subjects, sample size, diagnostic criteria, inclusion and exclusion criteria, randomization method, blinding, treatment and control measures, outcome indicators, and safety indicators.

#### 2.6.3 Literature Quality Assessment and Evidence Analysis

The quality of guidelines will be assessed using the AGREE II (Appraisal of Guidelines for Research and Evaluation II) tool. Meta‐analyses will be evaluated using the AMSTAR (A Measurement Tool to Assess Systematic Reviews, AMSTAR) scale. Cohort studies and case‐control studies will be assessed using the Newcastle‐Ottawa Scale (NOS). The Cochrane Risk of Bias tool [11] was employed to assess the methodological quality of randomized controlled trials (RCTs). Seven types of bias were assessed: random sequence generation, allocation concealment, blinding of participants and personnel, blinding of outcome assessment, completeness of outcome data, selective reporting, and other biases. The risk of bias was categorized as “low risk,” “unclear,” or “high risk,” and a risk of bias assessment chart was generated.

RevMan 5.3 software was utilized to integrate and analyze the data from the original studies. This software facilitated data entry and analysis, followed by generating corresponding charts, such as the risk of bias assessment table, a risk of bias summary graph, and a meta‐analysis forest plot.

#### 2.6.4 Evidence Quality Evaluation and Recommendation Standards

The Grading of Recommendations, Assessment, Development, and Evaluation (GRADE) system was used to summarize and evaluate the quality of the included evidence [12]. According to GRADE, evidence quality was categorized into four levels: high, moderate, low, and very low. Based on the strength of recommendations rated by the GRADE system and expert opinions, recommendations were formed for consensus items with supporting evidence. For items without supporting evidence, consensus suggestions were made [13].

### 2.7 Establishment of Recommendations

Based on the recommendations and consensus suggestions rated by the GRADE system, the first round of expert questionnaires was developed and sent via WeChat/SMS to pediatric TCM and Western medicine experts nationwide selected according to the Delphi method. The responses were collected and statistically analyzed to calculate the proportions of different levels of recommendations (strong recommendation + weak recommendation) for each item. When the recommendation proportion was ≥70%, consensus was achieved, and no further voting was necessary. When the proportion of strong recommendations was ≥70%, the recommendation level was classified as strong; when the proportion of strong recommendations was <70% but the combined proportion of strong + weak recommendations was ≥70%, the recommendation level was classified as weak. The recommendations from the first round of questionnaires were summarized and combined with written feedback from the experts to formulate the second round of questionnaires. This questionnaire was subsequently sent to the experts who responded to the first round, the results of which were collected and statistically analyzed to establish the final recommendations.

### 2.8 Compilation of the Consensus Document

On March 17, 2024, the project group held a consensus meeting with TCM and Western medicine experts. After thorough discussions and repeated deliberations based on their clinical experience, the participating experts finalized the consensus recommendations and their corresponding GRADE recommendation levels, thus enriching the details of these recommendations. Following revisions to the descriptions of specific recommendations based on the feedback from TCM and Western medicine experts, the initial draft of the consensus was formed. The guideline manuscript was subsequently written according to the Reporting Items for Practice Guidelines in Healthcare (RIGHT) checklist [14]. The following mainly presents the relevant issues and recommended opinions in the field of integrated traditional Chinese and Westrn medicine treatment.

## 3 Integrated Traditional Chinese and Western Medicine Treatment

### 3.1 Time to Start Drug Therapy in Pediatric Patients With TD

Practicing psychoeducation and/or behavioral interventions combined with acupoint massage before the initiation of drug therapy is recommended for pediatric patients with mild transient TD with a total Yale Global Tic Severity Scale (YGTSS) score of <19 points, a course of disease <1 year, and single TD symptoms that do not lead to impaired social functions; however, drug therapy may be initiated if the above symptoms are not markedly improved after intervention for >3 months. (weak recommendation, expert consensus)

Drug therapy may be initiated if the total YGTSS score is not <20 points [15, 16], if symptoms persist for >3 months, and if the pediatric patients and their family members demand immediate treatment of the patients. (strong recommendation, expert consensus)

Pediatric patients, whose manifestations lead to impaired social functions and compromise their daily life and school and social activities, may be treated with TCM or Western medicine alone. They may also be treated with integrated traditional Chinese and Western medicine (ITCWM) in the absence of a satisfactory therapeutic effect. (strong recommendation, expert consensus)

### 3.2 Indications of Using Traditional Chinese Medicine Alone to Treat TD in Pediatric Patients

In the previous 43 RCTs [15, 16, 17, 18, 19, 20, 21, 22, 23, 24, 25, 26, 27, 28, 29, 30, 31, 32, 33, 34, 35, 36, 37, 38, 39, 40, 41, 42, 43, 44, 45, 46, 47, 48, 49, 50, 51, 52, 53, 54, 55, 56, 57], treatment with Western medicine typically began at the age of 6 years, and treatment with TCM began at the age of 4 years. Therefore, TCM alone is recommended for use in pediatric patients aged 6 years or younger. (strong recommendation, expert consensus)

Pediatric patients with mild to moderate TD that are not complicated by comorbidities [58, 59, 60]. (weak recommendation, expert consensus)

Pediatric patients with TD who have been satisfactorily treated with TCM and who relapsed after drug withdrawal. (strong recommendation, expert consensus)

Pediatric patients if the patients or their family members express concerns about or deny the use of Western medicine or if the patients cannot tolerate Western medicine therapy. (strong recommendation, expert consensus)

### 3.3 Indications for Using ITCWM to Treat TD in Pediatric Patients

Pediatric patients with moderate to severe TD who meet any of the following criteria can be directly treated with ITCWM: (1) disease course of >3 years [61]; (2) presence of two or more comorbidities [62]; (3) pediatric patients who are at high risk of suffering from tic episodes induced or exacerbated by the family living environment [63, 64] and whose symptoms cannot be improved in the short term. (strong recommendation, expert consensus)

In the presence of the following cases, Western medicine can be used in addition to TCM: (1) tic episodes are ineffectively controlled (the YGTSS score is improved by <50%) or exacerbated 3 months after TCM therapy [65]; (2) TDs are improved, but comorbidities are not improved or are exacerbated 3 months after TCM therapy. (strong recommendation, expert consensus)

For refractory TD or TD that recur repeatedly during a period of general reduction or after discontinuation of Western medicine treatment, TCM can be used as a combined therapy alongside Western medicine treatment. (strong recommendation, expert consensus)

### 3.4 ITCWM Modalities for Treating TD in Pediatric Patients

ITCWM, which constitutes the use of TCM therapies (e.g., Chinese patent medication, TCM decoctions, auricular point sticking technique, and acupuncture) combined with psycho‐behavioral therapy and Western medicine therapies, is superior to TCM or Western medicine alone in treating TD in pediatric patients.

#### 3.4.1 TCM Decoctions Combined With Western Medicine

**Table:** 

Recommendations	Method	Evidence description
Lonicera and forsythia powder combined with haloperidol is recommended for use in pediatric patients with TS (reddish tongue, tightness, and discomfort in the neck, and retropharyngeal folliculosis) as it can improve the symptoms of vocal and motor tics. (weak recommendation, Grade C evidence)	Lonicera and forsythia powder consists of flos lonicerae, fructus forsythiae suspensae, herba menthae haplocalycis, herba schizonepetae tenuifoliae, semen sojae praeparatum, fructus arctii, radix platycodi, herba lophatheri, rhizoma phragmitis, and radix glycyrrhizae. The powder is decocted in water for oral administration; one dose is administered twice daily.	In a previous study, 126 pediatric patients with TS aged 2–14 years were clinically treated for 3 months [66, 67], and the response rate was greater (relative risk [RR] = 1.26 [1.08, 1.48], *p* < 0.05), and the YGTSS score was noticeably lower (mean difference [MD] = –6.3 [–6.67, –5.93], *p* < 0.05) in the combination therapy group than in the haloperidol monotherapy group.
Modified Chaihu Shugan powder combined with aripiprazole is recommended for use in pediatric patients with chronic motor or vocal TD (syndrome of liver depression and spleen deficiency), as it can improve the clinical efficacy, tic symptoms, and incidence of adverse reactions. (weak recommendation, Grade C evidence)	Modified Chaihu Shugan powder (formula granules) consists of fructus aurantii submaturus, radix glycyrrhizae preparata, radix aucklandiae, rhizoma chanxiong, fructus crataegi preparata, semen cassiae obtusifoliae, ramulus uncariae rhynchophyllae cum uncis, spica prunellae vulgaris, radix bupleuri chinensis, and radix paeoniae alba. The powder is dissolved in 80 mL boiled water for separate administration in the morning and evening, with one dose administered per day.	In a previous study, pediatric patients with TD complicated by the syndrome of liver depression and spleen deficiency aged 2–14 years were treated for 3 months [68]. The results indicated that the response rate was markedly greater (RR = 1.29 [1.09, 1.52], *p* < 0.05), and the incidence of adverse reactions was lower (RR = 0.09 [0.02, 0.37], *p* < 0.05) in the combination therapy group than in the aripiprazole monotherapy group.
Gouteng Dingfeng decoction combined with tiapride hydrochloride is recommended for use in pediatric patients with TS (syndrome of liver yang hyperactivity and stirring wind, characterized by frequent and intense tics, noticeable facial tics, and/or occasional yelling with a high‐pitched voice, accompanied by vexation, agitation, irascibility, dizziness, and headache), as it can improve the clinical efficacy and tic symptoms. (weak recommendation, Grade D evidence)	Gouteng Dingfeng decoction comprises ramulus uncariae rhynchophyllae cum uncis, fructus toosendan, concha haliotidis, fructus lycii, tribulus terrestris, scorpio, periostracum cryptotympanae, pheretima aspergillum, rehmannia dride rhizome, prepared rhizome of rehmannia, and radix glycyrrhizae. It is decocted in water for oral administration after meals, with one dose administered twice per day.	In a previous study, pediatric patients with TS complicated by the syndrome of liver hyperactivity and stirring wind aged 4–18 years were treated for 8 weeks [69]. The results suggested that the response rate was apparently greater (RR = 1.2 [1.00, 1.44], *p* = 0.05) and the YGTSS score was lower (MD = –3.31 [–5.66, –0.96], *p* < 0.05) in the combination therapy group compared to the tiapride monotherapy group.

#### 3.4.2 Chinese Patent Medications Combined With Western Medicine/Psycho‐Behavioral Therapy

**Table:** 

Recommendations	Evidence description
Jiuwei Xifeng granules combined with tiapride are recommended for use in pediatric patients with TD (syndrome of kidney yin deficiency and internal stirring of liver wind), as it can improve tic symptoms and the quality of life. (weak recommendation, Grade C evidence)	An RCT [26] suggested that cure and marked response rates increased significantly (RR = 1.63 [1.02, 2.59], *p* < 0.05) and the total YGTSS score decreased remarkably (MD = –6.55 [–7.11, –5.39], *p* < 0.05) in 90 pediatric patients with TD after treatment for 8 weeks. Another RCT [27] reported that the total YGTSS score decreased considerably (MD = –5.87 [–11.19, –5.23], *p* < 0.05) after 89 pediatric patients with TD aged 4–15 years were treated for 24 weeks. In addition, the symptoms (e.g., feverish sensations in palms and soles, tidal fever and night sweats, and vertigo and tinnitus) and quality of life were markedly improved in the combination therapy group (*p* < 0.05).
Changma Xifeng tablets combined with clonidine transdermal patch/tiapride are recommended for use in pediatric patients with TD, as they can diminish the frequency of tics and improve the quality of life. (weak recommendation, Grade C evidence)	A previous RCT [70] showed that the response rate was notably greater (RR = 1.09 [1.01, 1.18], *p* < 0.05), the total YGTSS score and Toronto Extremity Salvage Score were noticeably lower (MD = –2.21 [–2.67, –1.75], *p* < 0.05) and (MD = –0.56 [–0.79, –0.33], *p* < 0.05), and the physical, psychological, social, cognitive, and emotional function scores were higher in the combination therapy group among 146 pediatric patients with TD after treatment for 1 month. Another RCT [71] indicated that the response rate was higher (RR = 1.21 [1.01, 1.43], *p* < 0.05), the total YGTSS score was lower (MD = –3.72 [–4.89, –2.55], *p* < 0.05), and the Pediatric Quality of Life Inventory Generic Core Scale score was higher (MD = 5.59 [1.9, 9.28], *p* < 0.05) in the combination therapy group compared to the tiapride monotherapy group among 102 pediatric patients with TS after treatment for 12 weeks.
Shaoma Zhijing granules combined with tiapride are recommended for use in pediatric patients with TD characterized by eye blinking, neck stretching, grinning, and shoulder shrugging as they can improve tic symptoms and reduce adverse reactions. (weak recommendation, Grade C evidence)	Three RCTs [72, 73, 74] indicated that the response rates were considerably higher (RR = 1.28 [1.14, 1.43], *I* ^2^ = 0%, *p* < 0.05), the total YGTSS score and motor and vocal tic scores were markedly lower (MD = –1.87 [–2.91, –0.83], *p* < 0.05), (MD = –5.81 [–6.96, –4.66], *I* ^2^ = 0%, *p* < 0.05), and (MD = –7.55 [–8.99, –6.11], *I* ^2^ = 69%, *p* < 0.05), and the incidence of adverse reactions was significantly lower (RR = 0.33 [0.19, 0.6], *I* ^2^ = 0%, *p* < 0.05) in the combination therapy group in 270 pediatric patients with TD aged 5–18 years who were clinically treated for 8–12 weeks.
Jing Ling oral liquid combined with tiapride is recommended for use in pediatric patients with TS (syndrome of deficiency of kidney yin and excess of liver yang) as it can reduce the number of muscle groups affected by tics, frequency and intensity of tics, and incidence of adverse reactions. (weak recommendation, Grade C evidence)	A previous RCT [75] reported that the combination therapy was superior to tiapride monotherapy in markedly improving the response rate (RR = 1.58 [1.18, 2.10], *p* < 0.05), number of muscle groups affected by tics and frequency of tics (MD = –2.25 [–2.43, –2.07], *p* < 0.05), (MD = –1.74 [–1.89, –1.59], *p* < 0.05), and (MD = –1.54 [–1.68, –1.40], *p* < 0.05), and the incidence of adverse reactions (RR = 0.11 [0.03, 0.44], *p* < 0.05) in 62 pediatric patients with TS treated for 4 months.
Xiaoer Zhili syrup combined with tiapride/topiramate is recommended for use in pediatric patients with TD (syndrome of stirring of wind due to yin deficiency, characterized by frowning, blinking, grinning, head shaking, shoulder shrugging, hand flapping and kicking, and uttering strange sounds, obscenities, and profanities) as it can improve tic symptoms. (weak recommendation, Grade C evidence)	An RCT [76] reported that after 119 pediatric patients with TD complicated by a syndrome of stirring of wind due to yin deficiency were treated with Xiaoer Zhili syrup combined with tiapride for 12 weeks, the control rate and marked response rate were noticeably greater in the combination therapy group than in the Xiaoer Zhili Syrup monotherapy group (RR = 1.31 [1.01, 1.69], *p* < 0.05). The control and marked response rates did not differ between the combination therapy and tiapride monotherapy groups (RR = 1.31 [0.95, 1.8], *p* > 0.05). The control and marked response rates for TCM syndrome were apparently greater in the combination therapy group as compared to the Xiaoer Zhili syrup monotherapy group (RR = 1.28 [1.04, 1.57], *p* < 0.05). Moreover, they did not differ between the combination therapy and tiapride monotherapy groups (RR = 1.28 [0.98, 1.67], *p* > 0.05). A different RCT [77] reported that the response rates did not differ significantly between the combination therapy and topiramate monotherapy groups (RR = 1.07 [0.95, 1.2], *p* > 0.05) and the total YGTSS score decreased drastically (MD = –4.77 [–7.77, –1.77], *p* < 0.05) in 168 pediatric patients with TD treated for 8 weeks with Xiaoer Zhili syrup combined with topiramate.
Dimu Ningshen oral liquid combined with tiapride hydrochloride is recommended for use in pediatric patients with TS (syndrome of liver and kidney yin deficiency and liver yang hyperactivity) as it can improve the clinical efficacy, tic symptoms, and incidence of adverse reactions. (weak recommendation, Grade C evidence)	A previous RCT [78] reported that the response rate was markedly greater (RR = 1.36 [1.05, 1.76], *p* < 0.05), the total YGTSS score was remarkably lower (MD = –7.15 [–11.02, –3.28], *p* < 0.05), and the incidence of adverse reactions was lower (RR = 0.38 [0.15, 0.99], *p* = 0.05) in the combination therapy group compared to the tiapride monotherapy group among 90 pediatric patients with TS.
Changma Xifeng tablets combined with psycho‐behavioural therapy are recommended for use in pediatric patients with TS (syndrome of liver yang hyperactivity and stirring wind, characterized by tics accompanied by vexation, agitation, irascibility, dizziness, headache, restless insomnia, and inattention) as they can improve tic symptoms. (weak recommendation, Grade D evidence)	In a previous study, 60 pediatric patients with TS aged 2–18 years were clinically treated [79]. The results demonstrated that the total YGTSS score was lower (MD = –8.50 [–10.84, –6.16], *p* < 0.05) and the recurrence rate determined 1 month after follow‐up was lower (*p* < 0.05) in the combination therapy group than in the Changma Xifeng tablet monotherapy group.

#### 3.4.3 External Therapy Combined With Western Medicine

**Table:** 

Recommendations	Method	Evidence description
Acupuncture combined with haloperidol is recommended for use in pediatric patients with TS as it can improve the symptoms of motor and vocal tics. (weak recommendation, Grade C evidence)	Acupuncture technique: The pediatric patient is instructed to maintain a sitting position. Then, the acupoints Baihui, Sishencong, Yintang, Shenting, Shuigou, Zusanli (bilateral), Sanyinjiao (bilateral), Taichong (bilateral), Neiguan (bilateral), and Shenmen (bilateral) are selected. The scalp needle is inserted at a 15° angle to the scalp, and the body needle is inserted perpendicular to the skin. The twirling technique is applied at a frequency of approximately 200 times/min, and the needles are retained for 60 min (once daily, six times weekly).	In a previous study, 70 pediatric patients with TS aged 6–16 years were clinically treated [80]. The results revealed that the response rate was greater (RR = 1.32 [1.05, 1.65], *p* < 0.05) and the motor and vocal tic scores were lower (MD = –2.17 [–2.88, –1.46], *p* < 0.05) and (MD = –1.86 [–2.49, –1.23], *p* < 0.05) in the combination therapy group than in the haloperidol monotherapy group.
Scalp acupuncture combined with tiapride is recommended for use in pediatric patients with TS (syndrome of liver hyperactivity and spleen deficiency and syndrome of wind and phlegm attacking the upper part, characterized by emaciation or puffiness, sallow complexion, selective eating or anorexia, loose stools, amnesia, restless insomnia, vexation, agitation, irascibility, and large mood swings) as it can significantly improve the disease severity and symptoms of motor and vocal tics. (weak recommendation, Grade D evidence)	Acupuncture technique: Acupoint selection: bilateral motor areas and chorea tremor control area (the upper two‐fifths of area two, parallel with and 1.5 cm anterior to the motor area line). Method of operation: 75% alcohol is employed for routine disinfection. A 30‐gauge 1.5‐inch disposable sterile filiform needle is twirled to approximately 1 inch at a 15° angle to the scalp. Afterward, the mild reinforcing‐reducing technique is applied; that is, the needle is twirled at more than 200 revolutions/min for 1–2 min to move qi through the meridians, and it is twirled thrice at an interval of 15 min and retained for 45 min. Acupuncture is performed once daily at week 1 and once every other day thereafter.	In a previous study, 158 pediatric patients with TS aged 3–15 years were clinically treated for 1 month [81] The results suggested that the total YGTSS score was significantly lower (MD = –2.42 [–3.85, –0.99], *p* < 0.05) and (MD = –2.91 [–4.39, –1.43], *p* < 0.05) after treatment with scalp acupuncture combined with tiapride group compared to the tiapride/scalp acupuncture monotherapy group. Moreover, the posttreatment response rates did not markedly differ between the groups (RR = 1.05 [0.92, 1.21], *p* > 0.05; and RR = 1.07 [0.93, 1.23], *p* > 0.05, respectively).
Auricular point sticking technique combined with tiapride is recommended for use in pediatric patients with moderate to severe TD (syndrome of liver yang hyperactivity and stirring wind, accompanied by vexation, agitation, irascibility, dizziness, headache or hypochondriac fullness, reddish urine and dry stools, white or thin and yellowish tongue coating, and stringy and bounding pulses) as it can improve the TD severity. (weak recommendation, Grade D evidence)	Auricular point sticking technique: The auricle is routinely disinfected. Then, the auricular cowherb seed plaster is firmly affixed to an auricular point to which gentle pressure is applied so that the patient experiences localized sensations of soreness, distension, numbness, and pain. The auricular point is pressed for 30 to 60 seconds at a time, three to five times per day. Specifically, pressing and kneading operations are performed with the thumb and index fingers along the auricular point. The auricular points on both ears are alternatively tapped and pressed 5 days per week.	In a previous study, 60 pediatric patients with moderate to severe TD aged 7–14 years were clinically treated for 4 weeks [82]. The results revealed that the response rates and the recurrence rates determined 8 weeks after treatment did not differ significantly between the auricular point sticking technique combined with the tiapride group and the tiapride monotherapy group (RR = 1.14 [0.85, 1.52], *p* > 0.05) and (RR = 0.47 [0.09, 2.53], *p* > 0.05). Moreover, the total YGTSS score decreased remarkably 4 weeks after treatment (MD = –4.53 [–8.88, –0.18], *p* < 0.05).

### 3.5 Ways of Integrating Traditional Chinese and Western Medicine to Treat Refractory Tic Disorders in Pediatric Patients

Pediatric patients with refractory TD can be treated with antiepileptic drugs (e.g., sodium valproate and clonazepam) in addition to Western medication for TD. In the presence of comorbidities, symptomatic medication should be selected based on the comorbidities. In the presence of comorbid attention deficit hyperactivity disorder (ADHD), tomoxetine hydrochloride or clonidine should be the preferred choice for concurrent use. In the presence of comorbid obsessive‐compulsive disorder, sertraline or fluvoxamine should be the preferred choice for concurrent use. Medication with different mechanisms of action should be selected for concurrent use with two or more Western medications for TD. For pediatric patients who received Western medication to treat TD, Western medication for concurrent use should be appropriately selected based on their responses to drug therapies (e.g., efficacy and adverse reactions). (strong recommendation, expert consensus)

TCM therapies, including Chinese patent medication, TCM decoction pieces, acupuncture, and Tui na, can be selected and administered based on the compliance of pediatric patients and their parents' preferences. TCM decoction is generally preferred. No unified criteria are currently available for TCM syndrome differentiation of refractory TD. Previous cluster analysis suggested that the syndrome of spleen deficiency and liver hyperactivity, the syndrome of phlegm fire disturbing the mind, and the syndrome of external wind induction (syndrome of wind and heat stirring the wind) were the most frequent in patients with refractory TD [83], and that syndromes can be differentiated, formulas can be selected, and medication can be prescribed based on clinical experience. Pediatric patients who have difficulty receiving TCM decoctions can be treated with Chinese patent medication; however, such medication will be effective only if prescribed based on syndrome differentiation and if it corresponds to the syndromes. In addition, pediatric patients who receive acupuncture can be concurrently treated with moxibustion to improve the therapeutic effect, whereas young pediatric patients who receive acupuncture can be concurrently treated with Tui na. (strong recommendation, expert consensus)

### 3.6 Ways of Integrating Traditional Chinese and Western Medicine to Treat TD Complicated by ADHD in Pediatric Patients

Treating TD complicated by ADHD in pediatric patients is highly challenging. The pathophysiological mechanism underlying the correlation between TD and ADHD remains unknown. Studies indicated that TD may be genetically correlated with ADHD. The findings of numerous studies revealed that an imbalance between inhibitory and excitatory signals in the central nervous system represents a common mechanism underlying TD and ADHD. Further, TD are characterized by dopaminergic hyperactivity, while ADHD is characterized by dopaminergic hypofunction. In this regard, conventional treatment options may control tic symptoms; however, they can hardly alleviate comorbid attention deficits and hyperactive symptoms. TCM therapy for TD may be combined with Western medicine therapy for ADHD in actual clinical practice; however, no such evidence is currently available.

Studies regarding TCM have shown that TD complicated by ADHD are associated with functional disorders of five viscera and hypoevolutism of “the sea of marrow.” [84] Moreover, they are closely associated with the brain, liver, spleen, and kidney. Based on the concept of wholism, homotherapy for heteropathy is employed to write out prescriptions based on syndrome differentiation. ITCWM is frequently applied to improve the clinical efficacy in clinical practice. In Western medicine, tomoxetine hydrochloride, clonidine, and methylphenidate hydrochloride may be considered appropriate. TCM therapies typically include Jing Ling oral liquid, Xiaoer Zhili syrup, and Xiaoer Huanglong granules, which can nourish the yin to suppress the yang and calm the mind to extinguish wind. The above therapies generally last for 3 to 6 months and can effectively improve tics, hyperactivity, attention deficits, and adaptive behaviors in pediatric patients [85, 86, 87]. The recommendations are detailed below.

**Table:** 

Recommendations	Method	Evidence description
Jingling oral liquid combined with haloperidol is recommended for use in pediatric patients with TS complicated by ADHD (syndrome of essence insufficiency and liver and kidney qi deficiency, characterized by limb tremors, spasm of the muscles and vessels, head shaking and shoulder shrugging, grimacing and eye blinking, inattention, hyperactivity and loquacity, impulsivity and willfulness, and learning disorder, accompanied by hectic cheeks, feverish sensations in the palms and soles, and restless insomnia) as it can noticeably reduce YGTSS and ADHD scores. (weak recommendation, Grade C evidence)	Dosage and administration: Haloperidol tablets are orally administered at the starting dose of 0.5–1.0 mg/day, once in the morning and once in the evening. Moreover, the dosage is administered with a time‐dependent increase at an increment of no more than 0.3 mg per time until the conditions are controlled or the dose reaches 4 mg/day. The treatment cycle is 3 months.	A three‐month clinical observation of 150 pediatric patients with TS complicated by ADHD [85] indicated that the total response rate was greater (RR = 1.4 [1.2, 1.62], *p* < 0.05) and the posttreatment scores of motor and vocal tics; hyperactivity and impulsivity; and attention deficits were lower (MD = –4.92 [–5.9, –3.94], *p* < 0.05), (MD = –5.39 [–5.89, –4.89], *p* < 0.05), (MD = –2.3 [–2.69, –1.91], *p* < 0.05), and (MD = –3.971 [–4.81, –3.13], *p* < 0.05) in the combination therapy group compared to the haloperidol monotherapy group. Moreover, the primary adverse reactions were somnolence; nausea and vomiting; and dizziness in the combination therapy group, and the incidences of adverse reactions did not differ between both groups (RR = 0.36 [0.12, 1.09], *p* > 0.05).
Xiaoer Zhili syrup combined with aripiprazole is recommended for use in pediatric patients with TD complicated by ADHD (syndrome of disharmony between the heart and kidney and syndrome of turbid phlegm obstructing orifices, characterized by limb tremor; occasional vertigo; grimacing and eye blinking; coprolalia; wheezing because of retention of phlegm in the throat; hyperactivity and dysphoria; distracted heart and mind; insomnia and amnesia; and tidal fever and night sweats) as it can markedly diminish the YGTSS and ADHD scores. (weak recommendation, Grade C evidence)	Dosage and administration: Aripiprazole is orally administered at a starting dose of 2.5 mg once daily, and the dose increases at an increment of 2.5 mg per week until it reaches 10 mg/day. The treatment cycle is 4 months.	A 4‐month clinical observation of 64 pediatric patients with TD complicated by ADHD [86] demonstrated that the total response rate was greater (RR = 1.29 [1.05, 1.59], *p* < 0.05) and the YGTSS and ADHD scores were significantly lower (MD = –4.03 [–5.66, –2.4], *p* < 0.05) and (MD = –3.01 [–4.73, –1.29], *p* < 0.05) in the combination therapy group than in the aripiprazole monotherapy group. In addition, the combination therapy was superior to aripiprazole monotherapy in improving the cluster of differentiation CD3, CD4, CD4/CD8, immunoglobulin G, and immunoglobulin A levels in peripheral blood and immune parameters and functions in pediatric patients.
Xiaoer Huanglong granules combined with tiapride is recommended for use in pediatric patients with TD complicated by ADHD (syndrome of yin deficiency and yang hyperactivity characterized by frequent tics; hyperactivity and restless movements; head shaking and shoulder shrugging; bellowing; willfulness; poor self‐control; distracted heart and mind; hastiness and irritability; and garrulity and loquacity, accompanied by dizziness; headache; night sweats; dry mouth and throat; and feverish sensations in the palms and soles), as it can notably improve the YGTSS and Conners child behavior scale (CCBS) scores. (weak recommendation, Grade D evidence)	Dosage and administration: Tiapride is orally administered at a dose of 25–50 mg each time, twice to three times per day, and the dose is subject to a discretionary increase, depending on the tolerability in pediatric patients. The treatment cycle is 6 months.	A 6‐month clinical observation of 80 pediatric patients with TD complicated by ADHD [87] revealed that the total response rate was considerably greater (RR = 1.23 [1.01, 1.51], *p* < 0.05) and the YGTSS and CCBS scores in months 1, 3, and 6 were noticeably lower (MD = –4.56 [–5.4, –3.72], *p* < 0.05) and (MD = –2.96 [–3.45, –2.47], *p* < 0.05) in the combination therapy group than in the tiapride monotherapy group.

### 3.7 Clinical Outcome Assessment Cycle and Method of ITCWM Treatment in Pediatric Patients With TD

According to 22 studies on the ITCWM treatment of TD [26, 29, 66, 67, 68, 69, 70, 71, 72, 73, 74, 75, 76, 77, 78, 79, 80, 81, 82, 85, 86, 87], the major clinical outcome evaluation parameters included the YGTSS score, incidence of adverse reactions, and TCM syndrome integral. Moreover, most assessment cycles were 4–12 weeks. As no evidence that pertains to the time to switch medication during ITCWM treatment is available, it is advisable to (1) continue the maintenance treatment in pediatric patients with improved conditions based on evaluation results until the conditions are completely controlled and then reduce and discontinue the medication and (2) re‐assess the clinical outcome and adjust the treatment regimen accordingly in pediatric patients who have no response. (strong recommendation, expert consensus)

### 3.8 Matters Meriting Attention for ITCWM Treatment of TD in Pediatric Patients

(1) Selection of combination therapy regimen: Integrating TCWM should follow the principle of complementing each other's strengths. Western medicine plays a dominant role in rapidly resolving tics, while TCM is uniquely advantageous in improving biased constitution, regulating homeostasis, and producing minimal side effects based on eliminating pathogenic wind to arrest convulsion. ITCWM can remedy the shortcomings of TCWM. In addition, ITCWM treatments should be appropriately selected, and an individualized ITCWM treatment regimen should be established for pediatric patients based on an assessment of efficacy‐influencing factors [60], past history, comorbidities, medication history, and predisposing factors. (strong recommendation, expert consensus)

(2) Treatment compliance and safety: The parents of pediatric patients should be informed of the dosage form, method of administration, clinical efficacy, treatment cycle, and potential adverse drug reactions before a prescription is provided. Considering the acceptability of children and their parents’ preferences, ITCWM treatment options should be flexibly selected and promptly adjusted [88]. In the presence of combination therapy, safety parameters (e.g., hepatic and renal functions) should be examined once every 3 months [89]. In case of adverse reactions, medication should be adjusted or discontinued as directed by a physician. (strong recommendation, expert consensus)

(3) Method of administration: ITCWM medications should be administered at an interval of 30 min. Western medication, which induces adverse reactions (e.g., somnolence) compromising daytime activities in pediatric patients, is recommended for use in the evening. Pediatric patients with TD, whose symptoms are severe in the daytime and disappear after sleep initiation, may be administered two‐thirds of TCM medication in the morning and one‐third in the evening. TCM medication should preferably be administered twice per day and not three times per day whenever possible to boost treatment compliance. (weak recommendation, expert consensus)

### 3.9 Modalities for Reducing and Discontinuing ITCWM Medications for Treating TD in Pediatric Patients

Principle of medication reduction and discontinuation: Pediatric patients who meet the criterion for medication reduction (i.e., symptoms are controlled after maintenance treatment [90]) can enter the period of medication reduction and discontinuation. The principle of medication reduction is that reducing TCM medication is preceded by reducing Western medication. Reducing TCM medication may be followed by reducing Western medication for pediatric patients who have difficulty receiving TCM decoction pieces/Chinese patent medication. (strong recommendation, expert consensus)

Method of medication reduction and discontinuation: TCM medications should be maintained at their original doses during Western medication reduction and discontinuation. Western medication should be reduced in dosage and discontinued in a stepwise manner. For pediatric patients receiving two or more Western medications, the first‐initiated Western medication should be reduced and discontinued before the later‐initiated one(s).

The reduction in dosage and discontinuation period of each Western medicine is 1–3 months [90]. Western medication is reduced by one‐fourth once every 2 weeks, and it is alternately reduced and discontinued in the morning and evening. The reduction frequency of Western medication may be adjusted if necessary. (strong recommendation, expert consensus)

With respect to TCM medication reduction and discontinuation, discontinuing acupuncture is followed by reducing TCM decoction pieces and then Chinese patent medication. The reduction and discontinuation period of TCM decoction compounds should not be shorter than 2 months [58, 91, 92, 93]. TCM decoction compounds should be reduced and discontinued in order of one dose every 2 days and one dose every 3 days. They should be reduced for 1–2 months without any recurrence of conditions before the next course of reduction commences. Such reduction should continue until the decoction compounds are discontinued [93]. (strong recommendation, expert consensus)

Matters meriting attention for medication reduction and discontinuation: The season in which a medication is reduced correlates with recurrence. In winter and spring, the period of medication reduction and discontinuation may be appropriately extended as pediatric patients typically experience recurrent or aggravated conditions because of respiratory infections. Reducing medication is not recommended during the school terms when senior middle and high school students prepare for entrance examinations or during adaptation to the beginning of a term. As pediatric patients experience low levels of academic stress in the summer holiday, reducing and discontinuing medication in summer is vital for lowering the recurrence rate [93, 94, 95, 96]. In this regard, reducing and discontinuing medication in summer are recommended. It is advisable to return to the previous step or restart treatment If symptoms recur or worsen in pediatric patients during medication reduction [90]. (strong recommendation, expert consensus)

### 3.10 Assessment and Management of Adverse Reactions in Pediatric Patients With TD During Treatment

As the course of treatment is typically long for TD, adverse drug reactions should be closely monitored in clinical practice. For pediatric patients with mild adverse reactions and symptoms, continued medication reception is required for observation. For pediatric patients who experience severe adverse reactions or cannot tolerate adverse reactions, discontinuing or switching medications is recommended. TCM medications containing toxic components (e.g., medicinal insects and minerals) should be administered with caution in a given dose range according to the *Pharmacopoeia of The People's Republic of China*, modern pharmacology studies, and the experience of renowned and experienced TCM doctors. (strong recommendation, expert consensus)

## 4 Prevention and Care

Attention should be directed to promoting publicity and education and gaining the cooperation of the parents of pediatric patients and schoolteachers. With respect to the management of pediatric patients, these patients should have restricted time of access to electronic devices, practice recreational and sports activities, develop hobbies and interests, maintain healthy living habits, and devote attention to healthy dietary and nutritional habits. In addition, they should avoid factors that may lead to aggravated conditions (e.g., colds, overstrain, and exertion). They should also avoid consuming allergy‐causing, lead‐contaminated, and high‐fat foods; coffee and carbonated beverages; chocolate; and tea. With respect to family guidance (e.g., guidance on communications among family members and guidance on adverse psychological states), all efforts should be undertaken to create a sound family environment and to assist physicians in providing treatments so that pediatric patients with TD can recover as quickly and safely as possible.

## 5 Limitations and Deficiencies

This consensus, established based on the comprehensive collection of clinical questions, extensive evidence retrieval, literature analysis, and expert discussions, has some limitations: (1) the existing evidence from literature on ITCWM treatment of TD in pediatric patients is scarce and insufficient; (2) as TD are chronic neurodevelopmental disorders requiring a long treatment cycle, the long‐term efficacy of ITCWM requires further investigation; (3) some questions are not included because of a lack of evidence and consensus during the investigation of clinical questions. Such questions will be addressed by conducting more studies in a normative manner.

## 6 Update Plan

This consensus is intended to be updated, and the guidelines will be further developed in 2–3 years. The updates hinge on the presence of any new relevant evidence after the publication of this consensus, the impact of evidence changes on consensus recommendations, and the presence of changes in the strength of consensus recommendations. The update steps, which involve identifying new scientific evidence, assessing the necessity of updates, updating recommendations, and publishing updated consensus/guidelines, are implemented via literature research, surveys through questionnaires, and expert meetings.

## Conflicts of Interest

The authors declare no conflict of interest.

## References

[1] J. F. Leckman , “Tic Disorders,” BMJ (Clinical Research Ed) 344 (2012): d7659. 10.1136/bmj.d7659 22223834

[2] K. R. Müller‐Vahl , N. Szejko , C. Verdellen , et al., “European Clinical Guidelines for Tourette Syndrome and Other Tic Disorders: Summary Statement,” European Child & Adolescent Psychiatry 31, no. 3 (2022): 377–382. 34244849 10.1007/s00787-021-01832-4 PMC8940881

[3] T. Pringsheim , M. S. Okun , K. Müller‐Vahl , et al., “Practice Guideline Recommendations Summary: Treatment of Tics in People With Tourette Syndrome and Chronic Tic Disorders,” Neurology 92, no. 19 (2019): 896–906. 31061208 10.1212/WNL.0000000000007466 PMC6537133

[4] Z. S. Liu , Y. H. Cui , D. Sun , et al., “Current Status, Diagnosis, and Treatment Recommendation for Tic Disorders in China,” Frontiers in Psychiatry 11 (2020): 774. 32903695 10.3389/fpsyt.2020.00774 PMC7438753

[5] F. Li , Y. Cui , Y. Li , et al., “Prevalence of Mental Disorders in School Children and Adolescents in China: Diagnostic Data From Detailed Clinical Assessments of 17,524 Individuals,” Journal of Child Psychology and Psychiatry and Allied Disciplines 63, no. 1 (2022): 34–46. 34019305 10.1111/jcpp.13445

[6] Z. S. Liu , J. Qin , J. Q. Wang , et al., “Expert Consensus on the Diagnosis and Treatment of Tic Disorders (2017 Practical Edition),” Chinese Journal of Applied Clinical Pediatrics 32, no. 15 (2017): 1137–1140.

[7] R. Kurlan , Handbook of Tourette's Syndrome and Related Tic and Behavioral Disorders. 2nd ed (New York: Maecel Dekker, 2005).

[8] Y. Guo and Y. N. Guo , “Research Progress on Traditional Chinese Medicine Treatment of Tic Disorders,” Clinical Journal of Chinese Medicine 16, no. 12 (2024): 138–144.

[9] L. W. Zhang and J. Xie , “Systematic Review and Meta‐Analysis of the Clinical Efficacy of Integrated Chinese and Western Medicine in Treating Pediatric Tic Disorders,” Clinical Journal of Chinese Medicine 14, no. 03 (2022): 20–24.

[10] Y. Y. Yang , H. Huang , and C. S. Yang , “Quality Evaluation of Guidelines and Expert Consensus on Tic Disorders,” Journal of Pediatric Pharmacy 29, no. 8 (2023): 1–5.

[11] J. P. Higgins , D. G. Altman , P. C. Gøtzsche , et al., “The Cochrane Collaboration's Tool for Assessing Risk of Bias in Randomized Trials,” BMJ (Clinical Research Ed) 343 (2011): d5928. 10.1136/bmj.d5928 PMC3196245 22008217

[12] G. H. Guyatt , A. D. Oxman , G. E. Vist , et al., “GRADE: An Emerging Consensus on Rating Quality of Evidence and Strength of Recommendations,” BMJ (Clinical Research Ed) 336, no. 7650 (2008): 924–926. 10.1136/bmj.39489.470347.AD PMC2335261 18436948

[13] G. H. Guyatt , A. D. Oxman , R. Kunz , et al., “Going From Evidence to Recommendations,” BMJ (Clinical Research Ed) 336, no. 7652 (2008): 1049–1051. 10.1136/bmj.39493.646875.AE PMC2376019 18467413

[14] R. Xie , Y. Xia , Y. Chen , et al., “The RIGHT Extension Statement for Traditional Chinese Medicine: Development, Recommendations, and Explanation,” Pharmacological Research 160 (2020): 105178. 32889127 10.1016/j.phrs.2020.105178 PMC7462769

[15] V. Gabbay , J. S. Babb , R. G. Klein , et al., “A Double‐blind, Placebo‐Controlled Trial of ω‐3 Fatty Acids in Tourette's Disorder,” Pediatrics 129, no. 6 (2012): e1493–1500. 22585765 10.1542/peds.2011-3384 PMC3362909

[16] A Randomized, Double‐Blind, Placebo‐Controlled Trial Evaluating the Efficacy and Safety of Yi‐Gan San in Children and Adolescents With Tourette's Disorder [Internet]. 2018, https://clinicaltrials.gov/study/NCT03564132.

[17] F. Sallee , E. Kohegyi , J. Zhao , et al., “Randomized, Double‐Blind, Placebo‐Controlled Trial Demonstrates the Efficacy and Safety of Oral Aripiprazole for the Treatment of Tourette's Disorder in Children and Adolescents,” Journal of Child and Adolescent Psychopharmacology 27, no. 9 (2017): 771–781. 28686474 10.1089/cap.2016.0026 PMC5689110

[18] A. Ghanizadeh and A. Haghighi , “Aripiprazole Versus Risperidone for Treating Children and Adolescents With Tic Disorder: A Randomized Double Blind Clinical Trial,” Child Psychiatry and Human Development 45, no. 5 (2014): 596–603. 24343476 10.1007/s10578-013-0427-1

[19] R. Kurlan , G. Crespi , B. Coffey , et al., “A Multicenter Randomized Placebo‐Controlled Clinical Trial of pramipexole for Tourette's Syndrome,” Movement Disorders 27, no. 6 (2012): 775–778. 22407510 10.1002/mds.24919

[20] E. F. Hedderick , C. M. Morris , and H. S. Singer , “Double‐Blind, Crossover Study of Clonidine and Levetiracetam in Tourette Syndrome,” Pediatric Neurology 40, no. 6 (2009): 420–425. 19433274 10.1016/j.pediatrneurol.2008.12.014

[21] A Randomized, Double‐blind, Placebo‐Controlled Study of TEV‐50717 (Deutetrabenazine) for the Treatment of Tourette Syndrome in Children and Adolescents [Internet]. 2018, https://clinicaltrials.gov/study/NCT03452943.

[22] A Randomized, Double‐Blind, Placebo‐Controlled, Flexible Dose Study to Evaluate Efficacy and Safety of Pramipexole Immediate Release (0.125‐0.5 mg/Day) Versus Placebo for 6 Weeks in Children and Adolescents (Age 6–17 Inclusive) Diagnosed With Tourette Disorder According to DSM IV Criteria [Internet]. 2007, https://clinicaltrials.gov/study/NCT00558467.

[23] A Multicenter, Randomized, Double‐Blind, Flexible‐Dosed, Placebo‐Controlled, Parallel‐Group Clinical Trial Evaluating the Efficacy and Safety of Aripiprazole Oral Solution in Children and Adolescents With Tourette's Syndrome [Internet]. 2018, https://clinicaltrials.gov/study/NCT03487783.

[24] A Randomized, Double‐Blind, Placebo‐Controlled Study to Assess the Safety and Efficacy of NBI‐98854 in Pediatric Subjects With Tourette Syndrome [Internet]. 2016, https://clinicaltrials.gov/study/NCT02679079.

[25] A Phase 2b, Randomized, Double‐Blind, Placebo‐Controlled, Dose Optimization Study to Assess the Safety, Tolerability, and Efficacy of NBI‐98854 for the Treatment of Pediatric Subjects With Tourette Syndrome [Internet]. 2017, https://clinicaltrials.gov/study/NCT03325010.

[26] Q. J. Xiong , L. H. Ding , and S. Liu , “Study of Influence of Jiuweixifeng Granules Combined With Tiapride Hydrochloride Tablets on Neurological Function and Quality of Life of the Children With Tic Disorder,” Medical Forum 22, no. 11 (2018): 1448–1451.

[27] Y. Cao , “Efficacy Observation of Jintong Granules Combined With Tiapride Hydrochloride Tablets in the Treatment of Tic Disorders in Pediatric Patients With Kidney‐yin Deficiency and Internal Stirring of Liver Wind Pattern,” Modern Journal of Integrated Traditional Chinese and Western Medicine 27, no. 32 (2018): 3609–3612.

[28] S. X. Guo , S. Y. Hu , H. Liu , et al., “Efficacy and Safety of Choudongning Capsule in Children With Tourette's Syndrome of Spleen Deficiency and Phlegm Accumulation in Phase II Clinic,” Chinese Traditional and Herbal Drugs 49, no. 04 (2018): 891–896.

[29] C. Y. Du , S. Y. Hu , B. J. Zhao , et al., “Clinical Observation of Jiuwei Xifeng Granules in Treatment of Tic Disorder in Children With Kidney‐yin Deficiency and Liver‐Wind Stirring,” Drugs & Clinic 32, no. 04 (2017): 718–722.

[30] J. H. Li , R. Ma , S. Y. Hu , et al., “Jintong Keli for Treatment of Children Tic Disorder With Deficiency of Kidney Yin and Liver Wind Stirring up Internally Syndrome: A Randomized Double‐Blind Controlled Trial,” Journal of Traditional Chinese Medicine 57, no. 10 (2016): 860–863.

[31] N. Yang , R. Ma , S. Y. Hu , et al., “Efficacy and Safety of Choudongning Capsule (CDN) in Children With Tourette's Syndrome of Spleen Deficiency and Phlegm Accumulation,” China Journal of Chinese Materia Medica 41, no. 16 (2016): 3100–3106. 28920356 10.4268/cjcmm20161627

[32] S. Y. Hu , R. Ma , T. Tian , et al., “Phase III Clinical Research of Changma Xifeng Tablets in Treating Multiple Tics With Liver Wind and Sputum Stirring Internally,” Drugs & Clinic 29, no. 09 (2014): 1044–1049.

[33] R. Ma , S. Y. Hu , T. Tian , et al., “Zhidong Tablet and the Placebo Control Treatment of Tic Disorder Children Patients of Internal Disturbance of Gan‐Wind With Phlegm Syndrome: A Clinical Study,” Chinese Journal of Integrated Traditional and Western Medicine 34, no. 04 (2014): 426–430. 24812897

[34] J. Lv , Q. Q. Zhu , Y. P. Hao , et al., “A Double‐Blind, Double‐Dummy Clinical Study on the Efficacy of Choudongning Capsule in the Treatment of Tourette Syndrome in Pediatric Patients,” Global Traditional Chinese Medicine 6, no. S1 (2013): 85–86.

[35] Z. H. Ling , Clinical Study of 5‐Ling Granule in the Treatment of Tourette Syndrome in Pediatric Patients With Pattern of Wind Stirring due to Liver Hyperactivity and Pattern of Phlegm Fire Disturbing Upwards[D] (Nanjing University of Chinese Medicine, 2012).

[36] Chen YY , Lin X , Sheng LX , editors. Efficacy Observation of 5‐Ling Granule in the Treatment of Pediatric Tic Disorders (abstract). Collected Papers from the 9th Annual Academic Conference of Zhejiang Mental Health Association;2011.

[37] R. Ma , S. Y. Hu , X. W. Wei , et al., “Clinical Observation of Jintong Granules on Treating Tic Disorder in Children,” Global Traditional Chinese Medicine 3, no. 1 (2010): 31–34.

[38] L. Jin , R. Ma , S. Y. Hu , et al., “Clinical Research of Xifeng Zhidong Tablet in Treating Multiple Tics With Liver Wind and Sputum Stirring Internally,” Drugs & Clinic 25, no. 2 (2010): 148–151.

[39] Y. Q. Zhong , W. Z. Zhou , W. G. Hu , et al., “Randomized Double‐Blind Controlled Study on Treatment of Tic Disorders in Children With Transcutaneous Patch of Clonidine,” Chinese Journal of Pediatrics 45, no. 10 (2007): 785–787. 18211768

[40] W. D. Ji , Y. Li , N. LI , et al., “Olanzapine for Treatment of Tourette Syndrome: A Double‐Blind Randomized Controlled Trial,” Chinese Journal of Clinical Rehabilitation 9, no. 4 (2005): 66–68.

[41] D. Tao , T. Zhong , S. Ma , et al., “Randomized Controlled Clinical Trial Comparing the Efficacy and Tolerability of Aripiprazole and Sodium Valproate in the Treatment of Tourette Syndrome,” Annals of General Psychiatry 18 (2019): 24. 31624488 10.1186/s12991-019-0245-3 PMC6785853

[42] D. L. Gilbert , T. K. Murphy , J. Jankovic , et al., “Ecopipam, a D1 Receptor Antagonist, for Treatment of Tourette Syndrome in Children: A Randomized, Placebo‐controlled Crossover Study,” Movement Disorders 33, no. 8 (2018): 1272–1280. 30192018 10.1002/mds.27457

[43] T. K. Murphy , T. V. Fernandez , B. J. Coffey , et al., “Extended‐Release Guanfacine Does Not Show a Large Effect on Tic Severity in Children With Chronic Tic Disorders,” Journal of Child and Adolescent Psychopharmacology 27, no. 9 (2017): 762–770. 28723227 10.1089/cap.2017.0024

[44] M. H. Bloch , K. E. Panza , A. Yaffa , et al., “N‐Acetylcysteine in the Treatment of Pediatric Tourette Syndrome: Randomized, Double‐Blind, Placebo‐Controlled Add‐On Trial,” Journal of Child and Adolescent Psychopharmacology 26, no. 4 (2016): 327–334. 27027204 10.1089/cap.2015.0109 PMC6445198

[45] Y. Zheng , Z. J. Zhang , X. M. Han , et al., “A Proprietary Herbal Medicine (5‐Ling Granule) for Tourette Syndrome: A Randomized Controlled Trial,” Journal of Child Psychology and Psychiatry and Allied Disciplines 57, no. 1 (2016): 74–83. 26072932 10.1111/jcpp.12432

[46] M. E. Lemmon , M. Grados , T. Kline , et al., “Efficacy of Glutamate Modulators in Tic Suppression: A Double‐Blind, Randomized Control Trial of D‐serine and Riluzole in Tourette Syndrome,” Pediatric Neurology 52, no. 06 (2015): 629–634. 26002052 10.1016/j.pediatrneurol.2015.02.002 PMC4454293

[47] S. Z. Wang , F. Qi , J. Li , et al., “Effects of Chinese Herbal Medicine Ningdong Granule on Regulating Dopamine (DA)/Serotonin (5‐TH) and Gamma‐Amino Butyric Acid (GABA) in Patients With Tourette Syndrome,” BioScience Trends 6, no. 04 (2012): 212–218. 23006968 10.5582/bst.2012.v6.4.212

[48] Y. M. Awaad , “Levetiracetam in Tourette Syndrome: A Controlled Double‐Blind, Placebo Randomized Study,” European Journal of Neurology 17 (2010): 113.

[49] L. Zhao , A. Li , H. Lv , et al., “Traditional Chinese Medicine Ningdong Granule: The Beneficial Effects in Tourette's Disorder Randomized Double‐Blind Multicentre Placebo‐Controlled Clinical Trial of the Clonidine Adhesive Patch for the Treatment of Tic Disorders,” Journal of International Medical Research 38, no. 01 (2010): 169–175. 20233526 10.1177/147323001003800119

[50] R. Garcia‐Lopez , E. Perea‐Milla , C. R. Garcia , et al., “New Therapeutic Approach to Tourette Syndrome in Children Based on a Randomized Placebo‐Controlled Double‐blind Phase IV Study of the Effectiveness and Safety of Magnesium and Vitamin B6,” Trials 10 (2009): 16. 19284553 10.1186/1745-6215-10-16 PMC2660319

[51] Y. S. Du , H. F. Li , A. Vance , et al., “Randomized Double‐blind Multicentre Placebo‐Controlled Clinical Trial of the Clonidine Adhesive Patch for the Treatment of Tic Disorders,” Australian and New Zealand Journal of Psychiatry 42, no. 9 (2008): 807–813. 18696285 10.1080/00048670802277222

[52] C. L. Smith‐Hicks , D. D. Bridges , N. P. Paynter , et al., “A Double Blind Randomized Placebo Control Trial of Levetiracetam in Tourette Syndrome,” Movement Disorders 22, no. 12 (2007): 1764–1770. 17566124 10.1002/mds.21615

[53] R. Nicolson , B. Craven‐Thuss , J. Smith , et al., “A Randomized, Double‐Blind, Placebo‐Controlled Trial of Metoclopramide for the Treatment of Tourette's Disorder,” Journal of the American Academy of Child and Adolescent Psychiatry 44, no. 7 (2005): 640–646. 15968232 10.1097/01.chi.0000163279.39598.44

[54] X. L. Xie , D. H. Wu , X. Peng , et al., “Effects of Metoclopramide on the Symptoms as Well as Intelligence and Memory in Tourette Syndrome,” Chinese Journal of Clinical Rehabilitation 9, no. 40 (2005): 155–157.

[55] A Well‐Controlled, Fixed‐Dose Study of TEV‐50717 (Deutetrabenazine) for the Treatment of Tics Associated With Tourette Syndrome [Internet]. 2018, https://clinicaltrials.gov/study/NCT03571256.

[56] A Multicenter, Randomized, Double‐Blind, Placebo‐Controlled Study Evaluating the Safety and Efficacy of Flexible‐Dose Once‐Weekly Oral Aripiprazole in Children and Adolescents With Tourette's Disorder [Internet]. 2011, https://clinicaltrials.gov/study/NCT01418339.

[57] A Randomized, Placebo‐Controlled Trial to Evaluate the Long‐Term (ie, Maintenance) Efficacy of Oral Aripiprazole in the Treatment of Pediatric Subjects With Tourette's Disorder [Internet]. 2018, https://clinicaltrials.gov/study/NCT03661983.

[58] T. Zhao , Study on the Clinical Efficacy of Traditional Chinese Medicine Treatment Alone in 286 Pediatric Cases of Tic Disorders and Influencing Factors Associated With Tic Disorders (Tianjin: Tianjin University of Traditional Chinese Medicine, 2022).

[59] W. Y. Wang , Clinical Observation of Self‐Made Qingxin Xiegan Decoction in the Treatment of Tic Disorders in Pediatric Patients With Fire Hyperactivity of the Heart and Liver Pattern (Harbin: Heilongjiang University of Chinese Medicine, 2023).

[60] R. Guo , Clinical Efficacy Observation of Spleen‐strengthening and Liver‐Regulating Therapy in the Treatment of Tic Disorders in Patients With Spleen Deficiency and Liver Hyperactivity Pattern (Shenyang: Liaoning University of Traditional Chinese Medicine, 2023).

[61] F. He , H. H. Huang , Y. J. Qi , et al., “Factors Associated With Refractory Tourette's Syndrome,” Journal of Medical Research 49, no. 06 (2020): 99–104.

[62] Y. T. Tan , Study on Prognosis and Influencing Factors Associated With Tourette's Syndrome (Guangzhou: Guangzhou University of Chinese Medicine, 2012).

[63] X. M. Chen , M. L. Guo , and L. X. Yang , “Investigational Research on Factors Associated With the Onset of Tourette's Syndrome,” Chinese Pediatrics of Integrated Traditional and Western Medicine 3, no. 5 (2011): 385–387.

[64] L. X. Yang , X. M. Chen , Y. T. Tan , et al., “Analysis of Prognosis Influencing Factors of Children With Tourette's Syndrome,” Journal of Guangzhou University of Traditional Chinese Medicine 31, no. 6 (2014): 860–863.

[65] W. Y. Li , L. S. Deng , S. Mo , et al., “Clinical Observation of Integrated Traditional Chinese and Western Medicine Stage‐by‐Stage Therapy in the Treatment of Children's Tic Disorder,” Journal of Guangzhou University of Traditional Chinese Medicine 30, no. 2 (2013): 157–161.

[66] J. Qiu and R. J. Wang , “Clinical Study of Modified Yinqiao Powder in the Treatment of Tourette's Syndrome,” Journal of Hunan University of Chinese Medicine 37, no. 1 (2017): 62–64.

[67] L. Hu , “Clinical Research of Combining Traditional Chinese and Western Medicine for the Treatment of Children With Tourette's Syndrome,” Journal of Sichuan of Traditional Chinese Medicine 34, no. 10 (2016): 128–130.

[68] M. H. Cao , “Clinical Observation on Modified Chaihu Shugan San in Adjuvant Treatment of 102 Cases of Tic Disorder in Children With Stagnation of the Liver‐Qi and Deficiency of the Spleen Type,” Journal of Pediatrics of Traditional Chinese Medicine 15, no. 2 (2019): 71–73.

[69] J. Z. Zhang , Clinical Observation of Gouteng Dingfeng Decoction in the Treatment of Tourette's Syndrome in Patients With Pattern of Wind‐Stirring Due to Liver Hyperactivity (Zhejiang Chinese Medical University, 2019).

[70] L. L. Gai , “Clinical Efficacy of Changma Xifeng Tablet Combined With Clonidine Transdermal Patch in the Treatment of Children With Tic Disorder,” Chinese Journal of Modern Drug Application 17, no. 19 (2023): 153–156.

[71] L. Yin , R. Xu , H. Yang , et al., “Clinical Study of Changma Xifeng Tablets Combined With Tiapride in the Treatment of Multiple Tourette's Syndrome in Children,” Drugs & Clinic 38, no. 09 (2023): 2212–2217.

[72] A. H. Zhao , C. Zhou , and L. Cao , “Clinical Efficacy Analysis of Shaoma Zhijing Granules Combined With Tiapride in the Treatment of Children With Tic Disorder,” Systems Medicine 8, no. 05 (2023): 131–134.

[73] D. W. Kong , B. Li , Y. L. Du , et al., “Clinical Efficacy Observation of Shaoma Zhijing Granules Combined With Tiapride in the Treatment of Tic Disorders in Pediatric Patients,” Journal of China Prescription Drug 20, no. 08 (2022): 149–151.

[74] Y. X. Zhang , S. Y. Gao , S. Zhang , et al., “Clinical Study of Shaomazhijing Granules Combined With Tiapride in the Treatment of Tourette's Syndrome in Children,” Drugs & Clinic 37, no. 03 (2022): 521–525.

[75] R. B. Huang , S. H. Ge , and C. S. Fang , “Clinical Therapeutic Effect Analysis of Treating Pediatric Tourette's Disease by Combined Use of Tiapride and Jingling Oral Liquid,” China & Foreign Medical Treatment 36, no. 36 (2017): 137–139. 42.

[76] T. Y. Chen , H. M. Ruan , X. M. Han , et al., “Multicenter Observation of the Clinical Effects of Xiaoer Zhili Syrup in the Treatment of Children Tic Disorder With Syndrome of Stirring Wind due to Yin Deficiency,” China Journal of Traditional Chinese Medicine and Pharmacy 34, no. 12 (2019): 5983–5986.

[77] W. P. Xiang and L. Yao , “Clinical Observation of Xiaoer Zhili Syrup Combined With Topiramate in the Treatment of Children With Tic Disorder,” Drugs & Clinic 32, no. 03 (2017): 447–450.

[78] Z. H. Chen , X. F. Zhu , G. S. Yu , et al., “The Curative Effect of Dimuningshen Oral Liquid Combined With Tiopride in the Treatment of Children With Tourette's Disorder,” Zhejiang Clinical Medical Journal 22, no. 11 (2020): 1578–1579. 82.

[79] R. X. Li , Clinical Efficacy Observation of Changma Xifeng Tablets Combined With Psychological and Behavioral Interventions in the Treatment of Tourette's Syndrome in Patients With Pattern of Wind Stirring Due to Liver Hyperactivity (Shanxi University of Chinese Medicine, 2019).

[80] Y. Zhang , “Clinical Observation on 35 Cases of Children With Gilles de la Tourette's Syndrome Treated by Acupuncture Treatment Combined With Haloperidol,” Journal of Pediatrics of Traditional Chinese Medicine 15, no. 02 (2019): 74–77.

[81] D. Wang , Clinical Controlled Study of Scalp Acupuncture Combined With Tiapride in the Treatment of Tourette's Syndrome (Hubei University of Chinese Medicine, 2018).

[82] J. Y. Guan , Clinical Study of Auricular Point Sticking Combined With Tiapride Hydrochloride in the Treatment of Tic Disorders in School‐Age Pediatric Patients (Zhejiang Chinese Medical University, 2018).

[83] J. B. Xu , Study on the Distribution Characteristics of Traditional Chinese Medicine Syndrome and Medication Rule for Refractory Tic Disorders in Pediatric Patients (Tianjin University of Traditional Chinese Medicine, 2022).

[84] R. Ma and X. L. Zhang , “Discussion on Pathomechanism Hypothesis ADHD Caused by Growth Retardation of Spinal Marrow,” China Journal of Traditional Chinese Medicine and Pharmacy no. 8 (2008): 737–739.

[85] C. Gao , Y. L. Wu , Y. Q. Wei , et al., “Efficacy Observation of Jingling Oral Liquid Combined With Haloperidol in the Treatment of Tourette's Syndrome in Pediatric Patients,” Modern Journal of Integrated Traditional Chinese and Western Medicine 28, no. 2 (2019): 192–194.

[86] Y. Sun , H. P. Wang , L. F. Duan , et al., “Therapeutic Effect of Pediatric Intellectual Syrup Combined With Aripiprazole on Children With Tic Disorder and Its Effect on Immune Function,” Journal of Medical Information 32, no. 20 (2019): 139–141.

[87] J. Chen , “Efficacy Observation of Tiapride Combined With Xiaoer Huanglong Granules in the Treatment of Tic Disorders With Comorbid Attention Deficit Hyperactivity Disorder,” Journal of Modern Medicine & Health 36, no. 7 (2020): 1033–1035.

[88] C. S. Yang , Evidence‐Based Development of Clinical Medication Pathway and Adherence Management Strategies for Pediatric Tic Disorders (Sichuan University, 2021).

[89] Z. N. Xu , Y. Q. Zhao , S. Y. Chen , et al., “Advantage and Risk Analysis on Combination of Traditional Chinese Medicines and Western Medicines,” Chinese Traditional and Herbal Drugs 54, no. 2 (2023): 408–415.

[90] Q. Lu , D. Sun , and Z. Liu , “Interpretation of Expert Consensus for Diagnosis and Treatment of Tic Disorders in China,” Chinese Journal of Applied Clinical Pediatrics 36, no. 9 (2021): 647–653.

[91] R. Yang , Investigation of Prognosis and Long‐term Efficacy Evaluation of Jingxin Zhidong Formula‐Based Treatment Regimen in Adult Patients With Tic Disorders (Beijing University of Chinese Medicine, 2021).

[92] Y. X. Zhang , Study on Factors Associated With Tic Disorder Recurrence and the Effects of Traditional Chinese Medicine Treatment on this Recurrence (Tianjin University of Traditional Chinese Medicine, 2021).

[93] X. Wang , Study on Traditional Chinese Medicine Medication Reduction Strategies for Tic Disorders and Factors Associated With Tic Disorder Recurrence (Tianjin University of Traditional Chinese Medicine, 2023).

[94] X. M. Chen , Investigation of Factors Associated With Tourette's Syndrome and Traditional Chinese Medicine Etiology of Tourette's Syndrome: Clinical Integrated Traditional (Chinese and Western Medicine of Guangzhou University of Chinese Medicine, 2009).

[95] F. C. Zhang and L. Yang , “Clinical Experience and Insights on Syndrome Differentiation‐Based Treatment of Tourette's Syndrome,” Chinese Pediatrics of Integrated Traditional and Western Medicine 1, no. 2 (2009): 113–115.

[96] C. H. Wang and F. Han , “The Prognostic Analysis on the Treatment of 207 Cases of Tics‐Tourette's Syndrome With Chinese Medicine,” Journal of Hunan University of Chinese Medicine 32, no. 2 (2012): 64–65. 9.

